# Supplementary material for: Metformin and the Risk of Chronic Urticaria in Patients with Type 2 Diabetes
Source: Int J Environ Res Public Health. 2022 Sep 3;19(17):11045. doi: 10.3390/ijerph191711045 (PMC9517871; doi:10.3390/ijerph191711045)
Supplement: Supplementary file 1 [file ijerph-19-11045-s001.zip › ijerph-1877275-supplementary.pdf]

Table S1. Diseases and related ICD-9-CM, ICD-10-CM codes.

Disease	ICD-9-CM codes	ICD-10-CM codes
Type 2 diabetes	250.xx, except 250.x1 and 250.x3	E08-E13, except E10
Type 1 diabetes	250.x1 and 250.x3	E10
Chronic urticaria	708.1, 708.8, 708.9	L50.1, L50.6, L50.8, L50.9
Dialysis	V56.0, V56.8, V45.1	Z49.31, Z49.32, Z99.2
Hepatic failure	570, 572.2, 572.4, 572.8	K72.00, K72.01, K72.10, K72.11, K72.90, K76.2, K72.91, K76.7, K76.81
Vasculitis and allergic purpura	287	D69.1-D69.9
Overweight	278.02, 783.1, V85.2	R63.5
Obesity	278.00, 649.1, V77.8, V85.3	E66.09, E66.1, E66.8, E66.9, Z13.89
Severe obesity	278.01, 649.2, V45.86, V85.4	E66.01, E66.2
Smoking status	305.1, V15.82	F17, Z87.891
Alcohol-related disorders	291, 303, 305.0, 571.0-571.3, 790.3, V11.3, V79.1	F10, K70, R78.0, Z65.8
Hypertension	401–405	I10, I15
Dyslipidemia	272	E71.3, E75.2, E75.6, E77, E78
Coronary artery disease	410-414, 429	I20-I25
Chronic kidney disease	585, 586, 593.9, 791.0	N18, N19, R80.9
Stroke	430-438	I60-I69
Heart failure	428	I50

Peripheral arterial disease	440.0, 440.2-440.24, 440.3, 440.4, 443.81, 443.89, 443.9	I70.2, I70.92, I73.9, I75.0
Chronic obstructive pulmonary disease	491, 492, 496	J41-J44
Rheumatoid arthritis	714.0	M06.9
Systemic lupus erythematosus	710.0	M32.10
Liver cirrhosis	571.5, 571.2, 571.6	K70.2, K70.30, K70.31, K74.0, K74.5 K74.60, K74.69
Cancers	140-209	C00-C96
Psychosis	295, 297, 298	F20-F29
Depression	296.2, 296.3, 296.82, 296.99, 298.0,311	F32, F33
Dementia	290, 294.1, 331.0, 331.1, 331.2, 331.7, 331.8, 331.9	F01.50, F01.51, F02.80, F02.81, F03.90, F03.91, F05, G30, G31
Asthma	493	J45
Bacterial pneumonia	481, 482.41, 482.8, 486	J13, J18.1, J15.211, J15.6, J15.8, J15.5, A48.1, J18.8, J18.9

Table S2. Incidence rate and hazard ratio of chronic urticaria in patients with type 2 diabetes.

**Variables**	**Chronic urticaria**								
	**n**	**PY**	**IR**	**cHR**	**(95% CI)**	**p-value**	**aHR**	**(95% CI)**	**p-value**
Metformin non-user	496	142255	3.49	1	(reference)	-	1	(reference)	-
Metformin user	819	158517	5.17	1.49	(1.33, 1.66)	<0.001	1.56	(1.39, 1.74)	<0.001
**Sex**									
female	771	154627	4.99	1	(reference)	-	1	(reference)	-
male	544	146146	3.72	0.73	(0.65, 0.82)	<0.001	0.74	(0.66, 0.83)	<0.001
**Age**									
20-39	110	25346	4.34	1	(reference)	-	1	(reference)	-
40-59	568	131654	4.31	0.98	(0.8, 1.2)	0.8189	0.91	(0.74, 1.12)	0.3872
60-80	637	143773	4.43	0.98	(0.8, 1.2)	0.8556	0.84	(0.67, 1.05)	0.1278
**Comorbidities**									
Obesity	11	3436	3.2	0.68	(0.38, 1.24)	0.2085	0.62	(0.34, 1.13)	0.119
Smoking	15	3621	4.14	0.87	(0.52, 1.45)	0.5946	0.99	(0.59, 1.65)	0.9561
Alcohol disorders	33	7623	4.33	0.95	(0.67, 1.34)	0.7646	1.03	(0.71, 1.48)	0.8876
Hypertension	777	176609	4.4	0.99	(0.88, 1.1)	0.801	0.93	(0.82, 1.05)	0.2439
Dyslipidemia	769	168067	4.58	1.09	(0.98, 1.22)	0.1134	1.1	(0.98, 1.24)	0.1196
Coronary artery disease	455	87132	5.22	1.29	(1.15, 1.44)	<0.001	1.32	(1.15, 1.53)	<0.001
Stroke	177	45487	3.89	0.86	(0.73, 1)	0.0565	0.84	(0.71, 1.01)	0.059
Heart failure	84	16635	5.05	1.13	(0.91, 1.41)	0.275	1.03	(0.81, 1.31)	0.813
Peripheral arterial disease	48	8500	5.65	1.27	(0.95, 1.7)	0.1011	1.21	(0.9, 1.63)	0.1976
Chronic kidney disease	78	16562	4.71	1.03	(0.82, 1.3)	0.7938	1.18	(0.93, 1.49)	0.181
Chronic obstructive pulmonary disease	265	52054	5.09	1.19	(1.04, 1.37)	0.01	1.21	(1.05, 1.39)	0.0098
Rheumatoid arthritis	42	5966	7.04	1.61	(1.19, 2.2)	0.0023	1.46	(1.07, 2)	0.0159
Liver cirrhosis	28	5904	4.74	1.04	(0.72, 1.52)	0.8248	1.14	(0.77, 1.68)	0.504
Cancers	43	11537	3.73	0.81	(0.6, 1.1)	0.1755	0.92	(0.66, 1.27)	0.6105
Psychosis	15	5907	2.54	0.56	(0.34, 0.93)	0.0264	0.54	(0.32, 0.91)	0.0202
Depression	69	14244	4.84	1.09	(0.85, 1.38)	0.5084	1.06	(0.82, 1.36)	0.6584

Dementia	0								
**CCI**									
0	986	221646	4.45	1	(reference)	-	1	(reference)	-
1	166	37186	4.46	0.98	(0.83, 1.15)	0.7671	0.93	(0.78, 1.1)	0.3673
2+	163	41940	3.89	0.83	(0.7, 0.98)	0.0289	0.81	(0.66, 0.98)	0.0321
**DCSI**									
0	447	110146	4.06	1	(reference)	-	1	(reference)	-
1	267	60430	4.42	1.08	(0.93, 1.25)	0.338	0.98	(0.84, 1.15)	0.8161
2+	601	130296	4.62	1.11	(0.98, 1.25)	0.1063	0.99	(0.85, 1.16)	0.8856
**Medications**									
Sulfonylureas	485	83927	5.78	1.57	(1.4, 1.75)	<0.001	1.43	(1.26, 1.63)	<0.001
Thiazolidinedione	12	4594	2.61	0.59	(0.33, 1.03)	0.0649	0.54	(0.3, 0.95)	0.0337
Dipeptidyl peptidase-4 inhibitors	7	1461	4.79	0.94	(0.45, 1.97)	0.8613	1.13	(0.53, 2.39)	0.7524
Alpha-glucosidase inhibitors	37	8389	4.41	0.97	(0.7, 1.35)	0.8556	0.97	(0.7, 1.35)	0.8697
Oral anti-diabetic drugs number									
< 2	1287	294120	4.38	1	(reference)	-	1	(reference)	-
2-3	28			0.9	(0.62, 1.32)	0.6001	0.89	(0.61, 1.31)	0.551
> 3				5.61	(0.79, 39.87)	0.0847	6.99	(0.98, 50.09)	0.053
Insulin	355	65089	5.45405	1.34	(1.19, 1.51)	<0.001	1.21	(1.07, 1.38)	0.0032
Non-steroidal anti-inflammatory drugs	1177	246493	4.77498	1.96	(1.64, 2.33)	<0.001	1.88	(1.57, 2.25)	<0.001
Statins	314	72126	4.35352	0.96	(0.85, 1.09)	0.5447	0.87	(0.75, 1)	0.049
Aspirin	393	82017	4.7917	1.13	(1, 1.27)	0.0459	1.01	(0.88, 1.17)	0.8521

#: Adjusted HR estimated by the model including the variables of metformin, gender, age, comorbidities, and medications.

*: Per 1,000 person-year.

Abbreviation: CI, confidence interval; HR, hazard ratio; IR, incidence rate; T2D, type 2 diabetes mellitus.

Table S3. Incidences and hazard ratios of chronic urticaria for T2D patients with and without metformin use.

Variable	Non-metformin		Metformin						
	n	IR	n	IR	cHR	95%CI	aHR[a]	95%CI	Interaction p value
Sex									0.1455
Female	280	3.80118	491	6.06434	1.6	(1.38, 1.85)***	1.78	(1.53, 2.07)***	
Male	216	3.14897	328	4.22942	1.35	(1.14, 1.6)***	1.35	(1.13, 1.6)***	
Asthma									0.1283
No	421	3.41967	654	4.85159	1.42	(1.26, 1.61)***	1.49	(1.31, 1.68)***	
Yes	75	3.91771	165	6.95731	1.79	(1.37, 2.36)***	1.94	(1.47, 2.56)***	
Bacterial pneumonia									0.5117
No	474	3.5453	777	5.21387	1.48	(1.32, 1.65)***	1.55	(1.38, 1.73)***	
Yes	22	2.57095	42	4.42501	1.74	(1.04, 2.91)*	1.77	(1.05, 2.99)*	
Coronary artery disease									0.1425
No	324	3.11737	536	4.88576	1.57	(1.37, 1.8)***	1.66	(1.45, 1.91)***	
Yes	172	4.48834	283	5.79793	1.31	(1.08, 1.58)**	1.36	(1.12, 1.65)**	
Chronic obstructive pulmonary disease									0.5683
No	397	3.34245	653	5.0253	1.51	(1.33, 1.71)***	1.57	(1.39, 1.79)***	
Yes	99	4.21638	166	5.80938	1.39	(1.08, 1.78)**	1.49	(1.16, 1.93)**	
Rheumatoid arthritis									0.5786
No	482	3.45586	791	5.09229	1.48	(1.32, 1.66)***	1.54	(1.37, 1.73)***	
Yes	14	5.0321	28	8.79312	1.79	(0.94, 3.4)	1.9	(0.98, 3.7)	
Psychosis									0.3028
No	489	3.49988	811	5.22733	1.5	(1.34, 1.68)***	1.56	(1.4, 1.75)***	
Yes	7	2.76026	8	2.37327	0.86	(0.31, 2.37)	0.83	(0.28, 2.42)	
Charlson Comorbidity Index									0.4043
<2	453	3.57662	699	5.2884	1.48	(1.32, 1.67)***	1.55	(1.38, 1.75)***	
≧2	43	2.75656	120	4.55562	1.68	(1.19, 2.39)**	1.6	(1.12, 2.29)**	
Sulfonylureas									<0.001
No	455	3.5732	375	4.18957	1.14	(1, 1.31)	1.23	(1.07, 1.42)**	
Yes	41	2.74829	444	6.43394	2.54	(1.85, 3.5)***	2.07	(1.49, 2.86)***	
Insulin									0.0593
No	355	3.13977	605	4.93405	1.58	(1.38, 1.8)***	1.65	(1.45, 1.89)***	
Yes	141	4.8305	214	5.96106	1.24	(1, 1.53)*	1.31	(1.05, 1.63)*	
Non-steroidal anti-inflammatory drugs									0.6608
No	57	2.13631	81	2.93506	1.37	(0.98, 1.93)	1.41	(0.99, 2.02)	

Yes	439	3.79844	738	5.63704	1.49	(1.33, 1.68)***	1.59	(1.41, 1.79)***	
Statins									0.0917
No	400	3.60161	601	5.11118	1.42	(1.25, 1.61)***	1.51	(1.33, 1.72)***	
Yes	96	3.07753	218	5.32594	1.77	(1.39, 2.25)***	1.71	(1.33, 2.18)***	

[a] Adjusted HR estimated by the model including the variables of metformin, gender, age, comorbidities, and medications as shown in Table 1.

Abbreviation: CI, confidence interval; HR, hazard ratio; IR, incidence rate (Per 1000 person-years); T2D, type 2 diabetes.

* P<0.05, ** p<0.01, *** P<0.001
